# Supplementary material for: Are carbon emissions trading and green financial instruments synergistic? -Comprehensive quantitative research based on content analysis
Source: PLoS One. 2024 Mar 7;19(3):e0298601. doi: 10.1371/journal.pone.0298601 (PMC10919601; doi:10.1371/journal.pone.0298601)
Supplement: S1 Data — (ZIP) [file pone.0298601.s001.zip › Data/地级市Z_F工作报告.zip!/╣π╢1⁄2/╢1⁄2▌╕╩╨/└┤╫╘╣1⁄2╓┌║┼í╛┬φ┐╦╩2╛▌═°í┐_2012.docx]

各位代表：

现在，我代表市人民政府向大会报告过去五年的政府工作，并对今后五年和2012年工作提出建议，请予审议，并请政协各位委员和其他列席人员提出意见。

过去五年工作回顾

市第十四届人民代表大会第一次会议以来的五年，是东莞发展进程中不平凡的五年。五年来，在上级和市委的坚强领导下，全市上下深入落实科学发展观，全面实施《珠江三角洲地区改革发展规划纲要》，扎实推进经济社会双转型，较好地完成了市十四届人大一次会议确定的各项目标任务，东莞综合实力稳步增强，产业结构持续优化，人民生活不断改善，城市形象日益提升，获得全国文明城市、全国双拥模范城、国家园林城市、国家环保模范城市、中国制造业名城、中国十大最关爱民生城市等荣誉。

-----积极应对挑战，经济实力迈上了新的台阶。2008年以来，我市面临复杂多变的经济形势，经受了转型发展的考验和国际金融危机的冲击，在宏观环境较为困难的情况下，保持了经济的平稳健康发展。初步预计，全市生产总值从2006年的2628亿元增加到2011年的4735亿元，年均增长11.1%。来源于东莞的财政总收入、市财政一般预算收入、进出口总额、金融机构各项存款余额分别达到839亿元、313亿元、1347亿美元和6400亿元，是五年前的2.2倍、2.1倍、1.6倍和1.9倍。以落实规划纲要统领发展全局。深化“五个一体化”合作，加快深莞惠融合步伐，推动省、市重大项目实施，“四年大发展”各项目标任务完成良好。以一揽子举措应对金融危机。开展“稳定企业扎根发展”系列活动，设立多个10亿元专项资金帮扶企业。实行领导挂钩督导、审批绿色通道、要素资源倾斜等制度，促进重点项目建设提速，年均固定资产投资超过1000亿元。实施八大商贸促进工程，开展“莞货全国行”活动，社会消费品零售总额年均增长16.3%。以园区开发打造新增长极。松山湖升级为国家级高新区，工业总产值、税收年均分别增长40%和65%，台湾高科技园开园并引进了一批大型项目。虎门港建成3万吨级以上深水泊位12个，港口年吞吐量超过6000万吨。生态园基础设施和环境建设基本完成，升级为省级生态工业园区和循环经济示范园区。长安新区规划报批进展良好。各镇街工业集聚区加快整合提升。园区经济成为全市发展的重要增长极。以扶持镇村推动协调发展。提高镇街财政分成比例，落实贷款贴息政策，开展市内“双到”扶贫，实施生态补偿、镇村基础建设补助和公共管理支出补助，有效增强了基层发展的活力。2011年镇街平均可支配收入比2006年翻了1番，村组两级平均可支配收入达到3239万元。

-----深入调整结构，转型升级取得了突出成效。制定“1+26”政策体系，试点先行，加强引导，推动产业结构调整，实现了三大产业结构、经营模式、技术品牌、企业结构、市场结构、资源配套的优化。加工贸易转型步伐加快。在全国首创不停产转型模式，推动3500家来料加工企业转为独立法人企业，442家外资企业设立了研发机构，新增632家加工贸易企业开展品牌经营。全市外贸依存度从254%下降为187%，外企内销比重从26.4%上升到32.8%。东莞被确定为全国加工贸易转型升级试点城市。自主创新能力有效增强。市财政每年投入10亿元，2011年提高到20亿元，深入实施“科技东莞”工程。全市研发经费增长2.4倍，新增各类创新平台105个，企业参与各类标准修订223项。拥有国家高新技术企业415家、省级以上名牌名标528个，省创新科研团队数量和专利授权量居全省第二，社会创新意识和企业创新能力不断增强。招商引资和企业培育有新进步。深化与港台地区的经贸合作，拓展对日韩欧美的重点招商，五年累计实际利用外资128.4亿美元，引进超千万美元项目430宗、世界500强项目44宗。2008年以来实际引进内资694亿元、超亿元项目278宗。现代产业体系“四个30项目”、民营50强企业不断壮大，新增上市企业8家，年主营业务收入百亿元企业实现零的突破。现代服务业和战略性新兴产业加快发展。推动珠三角新兴物流城市建设，积极打造电博会、外博会、漫博会、台博会等品牌展会，推进“大麦客”等商业模式创新，金融、物流、会展、动漫等产业蓬勃发展，生产性服务业加快集聚，农业产业园建设扎实推进，三大产业比例从0.5：57.3：42.2调整为0.3：50.5：49.2。设立战略性新兴产业培育资金，启动五年倍增计划，新增全省战略性新兴产业基地3个。产业和劳动力“双转移”积极推进。市财政投入3亿元建设莞韶、莞惠产业转移园。全市新接收粤东西北地区劳动力23万人。

-----坚持环境取胜，城乡面貌得到了切实改善。五年引导全社会共投入城建资金2500多亿元，推动城市建设从中心城区向全市范围延伸，从基础设施向环境建设延伸，从拉开框架向完善功能延伸，强化了市区地位，优化了镇村形象，提升了城乡一体化水平，改善了水环境和空气质量，城市环境考核排名从全省第九升至第三。基础设施日趋完善。城际轨道和市内轻轨启动建设，莞深高速三期、莞惠高速、龙大高速、环城路、西部干道等高快速路建成通车，新建改造镇村联网路373公里，新建水利防灾减灾工程370项、110千伏及以上输变电工程76项、天然气管网790公里，城市承载力稳步增强。城市功能不断强化。“四院一中心”、第六水厂等投入使用，市篮球中心基本建成，中央商务区、中央商圈和生态休闲区规划建设全面启动，市区首位度不断提高，辐射带动作用更加突出。加快打造各镇街标志性片区，推进成熟社区、宜居社区和名村建设，镇街配套更加成熟。城乡环境持续优化。划定1104平方公里生态控制线范围，建成森林公园8个、乡村公园1031个、绿道638公里，森林覆盖率、建成区绿化率分别达到36.9%和45.4%。投入200多亿元建成34座污水处理厂及截污主干管网工程，污泥处理厂、医疗废物处理中心投入使用，运河综合整治取得实效，城镇生活污水处理率、生活垃圾无害化处理率分别达到77.3%和56.6%。圆满完成“十一五”节能减排任务。深入开展农村环境“五整治”，全面铺开“三旧”改造，促进了城市更新与城乡一体。城市管理水平提升。实行城管综合执法，推广“市场化+监理制”的市政管理模式，建立健全各项管理制度，推进生活垃圾分类试点，城市管理的制度化、专业化、社会化水平不断提高。

-----强化社会管理，有力维护了和谐稳定。落实维稳第一责任，着力提升社会管理水平，保障了社会稳定，增进了社会和谐。公共安全管理不断加强。每年将治安列为十件实事之首，加强治安防控，严打恶性和多发性犯罪，群众安全感增强，东莞获评全国社会治安综合治理优秀市。紧盯消防、交通、建筑、公共卫生、食品药品安全和产品质量等重点领域，深入推进大排查、大整治、大培训、大建设，全面提升安全生产管理水平。各类安全事故宗数、死亡人数、直接经济损失，比五年前分别下降33.9%、35.5%和41.3%。矛盾纠纷有效化解。完善信访调解网络，健全维稳综治体系，加强矛盾隐患排查，提升应急管理能力，强化劳动监察执法和劳资关系调解，群众信访总量下降19.8%，纠纷调处成功率达97.4%。新莞人服务管理逐步优化。成立新莞人服务管理机构，全面实施以居住证为载体的服务管理“一证通”制度，实行新莞人积分入户、子女积分入学政策，接受1.8万名新莞人积分入户、13.3万名新莞人子女入读义务教育阶段公办学校。新莞人参加各类社保超过2020万人次。

-----践行为民宗旨，民生事业实现了全面发展。把改善民生作为政府工作的出发点和落脚点，坚持每年办好十件实事，带动民生工作全面开展。五年来，市财政共投入688亿元用于民生发展，城市居民可支配收入和农村居民纯收入均增长56%。就业创业扶持有力。市财政每年安排10亿元实施“创业东莞”工程，累计培训劳动力20万人次，援助就业困难人员7万多人，城乡登记失业率控制在3.5%以内。向4400多人次提供小额创业贷款3.2亿元。最低工资标准提高到1100元。社保体系日益健全。农（居）民与职工基本养老实现统筹发展，退休金分别增长104%和55%。城乡基本医疗实现同等保障，医保最高支付限额提高到20万，医疗门诊报销70%。建立低保自然增长、临时物价补贴和高龄津贴制度，低保标准提高到440元，户籍困难家庭住房保障基本实现应保尽保。教育水平稳步提升。办学管理实现市镇统筹，省教育强镇实现全覆盖，义务教育实现全免费，高中阶段学校布局调整基本完成，职业教育、民办教育、高等教育加快发展，每万户籍人口大学入学人数全省第一。文化事业繁荣发展。推进图书馆、博物馆、文化广场建设，实现了村（社区）文化设施“五个有”全覆盖，完善了公共文化服务网络，创作了一批文艺精品，形成了一系列文化惠民活动品牌。设立5年共50亿元的专项资金，推动文化新城向文化名城跨越提升。卫生、体育等各项事业持续进步。推进以公益性为方向的新一轮医改，启动公立医院改革试点，建立基本药物制度，建成社区卫生服务机构390个。成功防控甲型H1N1流感、基孔肯雅热等疫情。年均人口自然增长率6.12‰。成功承办亚运会举重赛事，群众体育广泛开展，篮球运动屡获殊荣。积极做好残疾人康复、教育、就业服务。提前完成援建汶川映秀任务，大力援建新疆生产建设兵团农三师，对口援助西藏林芝、广西河池等地区，扎实推进韶关、云浮“双到”扶贫。国防双拥、外事侨务、海洋渔业、民族宗教、统计审计、气象防灾、档案方志、科普、打私、工青妇幼等工作取得新的进展。

-----不断改进作风，政府执行力进一步提高。深入转变政府职能，着力建设阳光、高效、为民的政府。改革创新力度不断加大。市政府工作部门从37个精简为32个，市直临时议事协调机构精简53%，行政审批事项精简33%。铺开简政强镇改革，向中心镇和市属园区下放经济社会管理权限500多项。开展村级体制改革试点，推进村民自治、经济发展、公共服务等职能分离。稳妥实施事业单位分类改革和人事制度改革。依法行政水平有效提高。加强法治政府建设，制定重大行政决策程序规定和行政处罚裁量标准，健全政府法律顾问、专家咨询论证、征求公众意见等制度。市长办公会向媒体开放，推行公共企事业单位办事公开，以网络、微博等方式强化了政群互动。自觉接受市人大、市政协及社会各界监督，五年来共办结人大代表、政协委员意见建议2576件，回复率和满意率均达100%。廉政和效能建设持续推进。严格落实廉政责任制，严控由财政出资举办的晚会、展览、庆典、论坛活动，严控楼堂馆所建设。建立市镇领导挂钩督导制度，将重要工作纳入行政问责范围，加强督促检查，狠抓工作落实。推进并联审批与网上审批，强化效能监察和电子政务建设，精简会议和文件。深入实施改进机关作风六项制度，组织明查暗访，每年开展“市民评机关”活动，优化了机关作风，提升了政府形象。

各位代表！过去五年，全市上下风雨兼程，励精图治，经受了严峻考验，战胜了重重困难，迈出了科学发展、转型发展的坚实步伐。这些成绩的取得，离不开中央、省委省政府和市委的正确领导，以及历届班子打下的坚实基础；离不开市人大、市政协与各民主党派的监督支持；离不开全市人民、驻莞部队、武警官兵的实干奋进，以及港澳台同胞、海外侨胞和国际友人的共同努力。在此，我代表市政府，向所有参与、支持和关心东莞经济社会发展的各界人士，表示衷心的感谢和崇高的敬意！

成绩令人振奋，经验弥足珍贵。实践充分证明：唯有坚持深入转型、加快升级，才能破解发展难题，转变发展方式。唯有坚持政府主导、市场主体，才能强化转型发展的科学导向，激发全社会的创造活力。唯有坚持产业强市、特色发展，才能发挥比较优势，巩固城市地位。唯有坚持关注基层、民生为本，才能促进协调发展，让改革成果更多更好地惠及老百姓。唯有坚持求真务实、强化执行，才能将上级和市委的决策部署，转化为推动东莞发展的有效举措。唯有坚持着眼大局、团结奋进，才能凝聚干事创业力量，推动东莞不断实现新的发展。

与此同时，我们也要清醒地看到经济社会发展面临的问题和存在的隐忧：一是产业结构调整任重道远。对传统发展模式的路径依赖仍然较强，产业核心竞争能力、自主创新能力和抗御风险能力仍待提升，新产业带动、大项目支撑力度不足。二是资源环境问题日益突出。土地、能源等要素供给越来越紧张，节能减排、生态建设和环境治理任务艰巨。三是社会管理压力依然较大。社会治安形势虽然有所好转，但与人民群众的期望仍有差距，安全事故还时有发生，矛盾排查调处与化解还不彻底，社会管理的体制机制和方法手段仍需优化。四是均衡发展水平尚待提升。个别镇村负债较重，集体经济稳健增长和持续增收面临考验，市内扶贫任务比较艰巨。五是城市管理模式仍较粗放。交通秩序、环境卫生、设施管养、内涝防治、违法建筑等方面的问题不可忽视，城市的精细化管理水平有待进一步提升。六是政府自身建设仍需加强。政府的创新意识、行政效能、服务水平等还有待改善，一些政府工作人员存在小富即安、小进则满、不求有功、但求无过的浮躁思想与懒散作风。对此，我们将以高度的使命感与紧迫感，认真有效加以解决。

今后五年奋斗目标和主要任务

未来五年，是面临重大挑战的发展转型期。从国际环境看，金融危机仍在延续，世界经济复苏的不稳定性、不确定性上升，东莞外向依存度较高，吸引外商投资、扩大外贸出口、稳定企业经营面临严峻考验。从国内环境看，全面开发开放步伐加快，区域竞争日益激烈，新开发城市大力招商引资，吸引了我市部分产业外移，而长三角、环渤海地区不少兄弟城市后来居上，在发展新兴产业方面形成了先发优势，我市产业结构调整面临严峻考验。从珠三角区域看，广州、深圳的中心城市地位突出，对我市的高端产业、人才等具有强大的磁吸效应，而惠州等周边城市成本和要素优势明显，对我市扩张期产业也具有较强的承接能力。如何找准自身定位，实施差异化发展战略，增强比较优势，集聚优势产业资源，是摆在我市面前的重大课题。从自身实际看，目前我市的人均收入已达到世界中等水平，传统的产业体系、发展方式无法为经济增长与企业增收提供足够动力，经济减速下行与社会矛盾凸显的趋势令人警惕，如果不能及时转变发展方式，以科技和制度创新注入新的动力，就无法跨越“中等收入陷阱”，甚至陷入发展停滞与矛盾冲突。内外困难倒逼转型，克服困难有赖转型，根本出路在于转型。

未来五年，是实现高水平崛起的战略机遇期。当前形势，危中有机。世界经济格局的深刻变化，加速了全球产业布局的调整；复杂严峻的外部环境，倒逼基层和企业加快转型升级的步伐。内需市场长期向好，国内新兴产业发展和创新成果转化不断加快，提供了巨大的增长空间。作为加工贸易转型升级、民政改革发展、“三旧”改造等全国、全省试点城市，我市拥有先行先试的宝贵机遇。改革开放30多年形成的对外开放优势、产业配套优势、城市环境优势，为未来发展奠定了良好基础。与广州、深圳等城市相比，我市在要素成本、配套成本、物流成本、行政成本、生活成本等方面，具有一定的综合优势，如果发挥利用得好，有利于吸引高端项目和人才。过去五年，东莞在结构调整、自主创新、体制改革等方面已经破了题、开了局。因此，只要我们抓住机遇，主动调整产业结构，加快转变发展方式，就有充分的信心实现高水平崛起。

实现高水平崛起，我们既要继承历届班子的宝贵经验与务实传统，也要与时俱进，锐意创新，不断开创工作新局面。未来五年工作中，我们一定要认真处理好五个方面的关系：

一是发展速度与发展质量的关系。坚持质量优先，速度服从质量，宁可在提升发展质量的过程中暂时减速，也不能追求低效益、高消耗、不可持续的速度。与此同时，要加快转型升级，培育新一轮增长的动力，释放内涵式发展的潜力，拓展高质量发展的空间，有效应对经济下行的压力，全面实现新的加速发展，通过几年的结构调整和扎实工作，让东莞在全省、全国重新领跑。

二是新兴产业与传统产业的关系。坚持“做大增量、调整存量、以增量扩张稀释存量”的思路，集中资源发展创新型产业尤其是战略性新兴产业，培育一批产值过千亿元的产业和超百亿元的企业，构建新的经济支柱与发展支撑。同时，要以工业化与信息化的融合改造提升传统产业，推动技术、设计、商业模式与分工体系的创新，加快转移淘汰高消耗、高污染、低效益的产业环节，提升核心竞争力与综合效益，更好地融入创新型经济体系，改善整个经济结构。

三是对外开放与对内开放的关系。外源型经济是我市的特色，必须通过深化对外合作、拓展海外市场、承接新兴产业转移等手段，不断保持和扩大先发优势。与此同时，必须深挖对内开放的潜力，大力开拓内需市场，积极引进国内大型央企、龙头民企、科技型中小企业和相关创新机构，使东莞成为国内外产业资源、创新资源高度集聚的城市。

四是市场调节与政府引导的关系。在经济转型的过程中，要把政府引导与市场机制结合起来，寓引导调节于主动服务之中，充分利用成本收益等价值规律，善于结合产业规划、功能区划、要素配置、资源价格、资助奖励等调控手段，有针对性地抓好项目发展与产业提升，对优质的初创型企业“雪中送炭”，加速孵化培育，对扩张期成长型企业“锦上添花”，助推做大做强，使市场调节更具导向性与时效性。

五是城市发展与群众生活的关系。城市发展既要服务于产业发展，更要服从于群众生活。要完善城市的设施、功能和布局，也要完善城市的环境、管理和服务，努力提供低成本、高品质的城市生活；要突出东莞的生态优势与自然特色，也要延续东莞的人文脉络与组团格局，努力塑造有特色、受认同的城市形象；要高水平普及教育、医疗、文化等公共服务，也要高水平满足群众在依法行政、社会公平、政府廉洁等方面的要求，让城市发展给全市人民带来全面的福祉。

未来五年，政府工作的总体要求是：全面贯彻市第十三次党代会精神，以科学发展为主题，以转变经济发展方式为主线，以加快转型升级、打造创新型经济为重点，以建设幸福东莞为宗旨，努力把东莞打造为全省科技和产业融合发展的示范区、结构调整和转型升级的样板区、创新社会管理的引领区，成为广东现代制造业名城、创新创业之城、生态文明之城、平安和谐之城，实现东莞的高水平崛起。

未来五年，全市发展的主要目标是：生产总值年均增长8%以上，人均生产总值年均增长7.5%以上，进出口总额年均增长8%以上，研究与开发经费支出占生产总值比例2.4%，城市居民人均可支配收入和农民人均纯收入年均增长8%以上，单位生产总值能耗降低19.5%，全市综合经济实力大幅提升，产业结构明显优化，城市竞争力和可持续发展能力显著增强；市民生活品质持续改善，做到人均收入更丰、就业机会更多、社会保障更强、文明程度更高、幸福感更强。

未来五年，政府工作的主要任务是：

一、致力打造创新型经济强市

创新型经济，就是以科技与产业融合为目标、以知识和人才为依托、以创新为动力、以新技术和新产品为着力点、以创新产业为标志的经济。发展创新型经济，是东莞实现高水平崛起的必然要求，是我市未来五年经济建设的核心任务，也是发展方式转变的核心内容。只有建立起创新型经济，推动经济发展从要素驱动、投资驱动向创新驱动转变，才能破除传统发展模式的路径依赖，防止落入“中等收入陷阱”；才能与韩国、新加坡、台湾等国家和地区一样，成功实现经济转型，步入良性发展轨道；才能拓展新的发展空间，引入新的发展动力，迎来新的增长拐点，实现新的发展格局。这要求我们在今后五年，把打造创新型经济作为经济建设的主攻方向，作为实现高水平崛起的主抓手，更坚定、更明确地把政府工作的重点放到产业建设上来，深入推进产业结构调整，推动东莞从产业大市向产业强市迈进。

主攻产业高端，构建创新型经济体系。以传统产业优势突出、战略性新兴产业形成规模、现代服务业支撑有力、都市型农业高效集约为目标，积极构建创新型经济体系。推动传统支柱产业加快拓展产业链条，抢占研发、设计、先进制造与营销等高端环节，提升产业竞争力与综合效益。加快战略性新兴产业的引进和培育，着力发展新一代信息技术、新一代互联网、新能源、新材料、节能环保、生物等产业，努力打造一批超千亿元战略性新兴产业集群。推动现代服务业加快聚集和拓展，突出发展与制造业配套的金融服务外包、物流仓储、研发设计、电子商务、文化创意等生产性服务业，积极发展总部经济、会展经济，打造珠三角新兴物流城市和金融强市。结合环境建设与生态修复，着力发展生产、生态、休闲、观光一体化的都市型农业。

夯实承载平台，优化创新型经济布局。完善园区承接创新型产业的载体功能，科学开发建设松山湖、虎门港、生态园、长安新区及各镇街工业集聚区，实施各镇街（园区）差异化发展战略。把大市区打造为金融中心、总部经济集聚中心和产业服务中心，加快建设以松山湖为龙头的园区经济带、以虎门港为核心的物流经济带、以主城区为核心的商贸经济带，加快形成现代服务业、高新技术产业、先进制造业、临港产业、都市型农业等集聚区，构建起“一个核心区、三大经济带、五大产业集聚区”的创新型产业格局。

集聚龙头项目，引领创新型经济发展。主动接受广州、深圳及港台等地辐射，积极承接高端产业转移，强化市级统筹，加强招商选资，在引进世界500强企业、大型跨国公司、大型央企和龙头民企上实现新突破。定向扶持一批优质企业做大做强，发挥大项目、大企业辐射效应，打造龙头项目配套企业群，力争带动一批现有产业集群整合提升、促进一批新兴产业集群有效形成。鼓励外资企业增资扩产和设立区域总部，协助拓展新兴市场和内销市场，增强产业植根性，提升开放型经济水平。

推进自主创新，增强创新型经济动力。完善“科技东莞”政策体系和服务体系，推动企业自主创新的资源投入与绩效提升，不断提高科技创新对经济增长的贡献率。集中财政专项资金力量，突出重点扶持一批关系城市未来发展的战略性、创新型产业项目。优化创新资源配置，建设和完善一批国家级、省级创新平台，加快突破一批共性和关键技术。深入实施人才东莞战略，加大创新科研团队和领军人才的引进培育力度，引导企业善待人才、用好人才，为创新型经济发展提供智力保障。大力扶持民营经济尤其是民营制造业发展，增强自主发展和抗御风险能力。

壮大现代金融，助推创新型经济提速。建设东莞金融商务区和松山湖金融改革创新服务区，在试点基础上推动科技金融、物流金融发展，加强莞台金融合作。扶持地方金融机构完善管理、整合壮大、扩张上市。有针对性地引进外资金融机构和跨境金融服务。加大优质企业上市培育力度，积极发展创业投资、产业基金等新型金融组织，引导形成多层次的投融资平台，促进金融、科技与产业融合发展，建设和完善现代金融体系。

二、致力建设宜居生态城市

按照“强心、宜居、内畅、外联”的思路，统筹布局各种公共设施和资源，建设更加宜居的生态城市。

打造中心突出、组团清晰的城市格局。以提高城市首位度为目标，高标准规划建设中央商务区、中央商圈和生态休闲区，努力把主城区打造成城市形象展示区和高级人才集聚区。合理布局全市性和片区性重大基础设施，把松山湖、虎门港、生态园、长安新区打造成产业升级引领区，把各镇打造成配套完善、各具特色的城市节点。促进布局集中、产业集聚、土地集约，遏制土地低效利用与城市无序扩张。

完善覆盖城乡、联接内外的基础网络。积极构建内聚外联的轨道交通网、一环六横六纵三连的高快速路网、四通八达的主干支次路网和便捷顺畅的公交体系，形成市内“半小时生活圈”、周边城市“一小时通勤圈”，全面融入珠三角交通一体化网络。健全城市供水、排水、治水体系，推进电信网、广播电视网、互联网三网融合，建设智慧城市和无线城市，完善城乡全覆盖的水电气设施和通讯网络。

塑造富有特色、现代品位的城市风格。加强重点区域、重点建筑的规划设计，加快城市更新改造，打造城市亮点和建筑精品。结合轨道交通的发展，沿线高起点规划、高标准建设一批城市综合体。借鉴世界先进城市经验，塑造人性化、生态化、特色化的公共环境，构筑错落有致、宽敞大气的城市空间。充分发挥我市江河湖库密集的资源禀赋和环境特色，加快推进沿江沿河的综合开发，彰显滨水特色，塑造生态形象。

营造生态良好、环境优美的人居环境。坚持环境建设与资源开发并举，生态保护与污染治理并重，加快森林公园、市镇和社区公园、绿道、慢行道等建设，推进绿化覆盖与生态控制。深入治理水系和大气污染，大力推行清洁生产，发展循环经济，推进节能减排。完善公共配套与休闲设施建设，加强城市精细化管理，使东莞成为最适宜创业与居住的城市之一。

三、致力提高市民幸福水平

建立健全幸福东莞指标体系，加快推进以改善民生为重点的社会建设，加强和创新社会管理，提高市民生活品质，提高社会净福利水平。

力保社会安定有序。坚持集中整治与长效管理相结合，积极构筑公共安全管理新模式，进一步完善社会治安立体防控体系、安全生产与食品药品安全监管体系、防灾减灾与应急管理体系，进一步完善利益协调、诉求表达、矛盾调处和权益保障机制，确保人民生命财产安全，确保矛盾纠纷有效化解，确保社会大局和谐稳定。

力推区域均衡发展。完善城乡统筹发展机制，逐步扩大公共财政对村级公共服务投入，减轻基层负担，促进城乡协调。帮助欠发达镇村加快发展，通过财税激励、项目支撑、结对帮扶等手段，增强欠发达镇村发展能力，确保发展速度不低于全市平均水平。创新集体经济发展模式，探索建立现代企业制度，开展多元化经营，实现集体资产保值增值。

力促发展成果共享。加强就业创业服务，健全工资正常增长机制。完善基本养老、基本医疗和最低生活保障制度，实现户籍人口社保全覆盖、新莞人参保率稳步提升、各类保障待遇逐步提高。完善住房保障机制，健全社会救助体系，加快发展慈善事业，加强残疾人保障服务。推进医药卫生体制改革，在解决看病难、看病贵问题上取得根本突破。促进基本公共服务均等化和优质化，稳步向新莞人群体覆盖，努力让每个市民都学有所教、劳有所得、病有所医、老有所养、住有所居。

四、致力提升文化软实力

以提升全民素质为核心，深化文化体制改革，优先发展教育事业，积极培育城市文明，全面提升城市软实力。

高水平建设文化名城。以满足人民精神文化需求为出发点和落脚点，用好文化专项资金，推进文化体制改革，实施文化惠民工程，保护历史街区和建筑，鼓励文艺精品创作，扶持文化产业发展，努力建设全国公共文化服务名城、国家历史文化名城、全国现代文化产业名城、岭南文化精品名城。

全方位打造教育高地。着眼于人的全面发展，整合教育资源，推动教育创新，力争在发展学前教育、提升义务教育优质均衡水平、促进职业教育与产业发展紧密结合、强化高等教育服务功能、推进特殊教育发展等方面实现新突破，建成省推进教育现代化先进市，教育综合竞争力达到国内先进地区水平。

深层次彰显文明形象。大力弘扬“海纳百川、厚德务实”的城市精神，深入开展文明创建活动，提升市民文明素质，提高城市文明程度。深化“六五”普法，强化全社会的法治意识。关爱弱势群体，推广志愿者服务，树立文明有序、和谐融洽的城市形象。

五、致力加强政府自身建设

深入转变政府职能，崇尚实干，激励创新，优化服务，依法行政，进一步改进机关作风，提升政府形象。

着力打造实干型政府。以提高效能为导向，树立奋发有为的意识，强化真抓实干的作风，全面推行“问题管理”模式，有效提升政府执行力。厉行勤俭节约，减少文山会海，强化工作问责，集中精力抓发展、抓落实，确保工作定一件、干一件、成一件。

着力打造创新型政府。深化简政强镇改革，加快形成有利于统筹发展、提升效能的行政管理体制。深化行政审批制度改革，进一步精简审批事项，优化办事流程。深化财政制度改革，积极构建公共财政体系。整合相关国有资产，建设政府投资平台，以金融创新手段筹集资金，推动城市建设与产业建设。积极培育发展社会组织，鼓励社会力量参与社会管理、提供公共服务。

着力打造服务型政府。牢固树立服务意识，高度重视社情民意，不断改进工作方法，强化督促指导和现场办公，及时解决社会关注的热点难点问题。实行“一站式”服务，加强政府电子政务建设，大力推广网上审批，严格执行服务承诺制，进一步提高服务水平，提升群众满意度。

着力打造法治型政府。坚持科学民主决策，落实重大行政决策程序，健全行政听证、社会公示、决策评估等制度。主动接受人大政协及社会各界监督。推进依法行政，深化政务公开，畅通诉求渠道，全面保障人民群众的知情权、表达权、参与权和监督权。完善行政执法责任制，落实行政过错责任追究制。扎实推进廉政建设和反腐败斗争，健全明查暗访长效机制，坚决整治损害群众利益的不正之风。

2012年工作安排

千里之行，始于足下；五年宏图，重在开局。2012年是我市推动高水平崛起的起步之年，是打造创新型经济的破题之年。政府工作的总体要求是：深入贯彻党的十七届六中全会、中央经济工作会议、省委十届十一次全会和市第十三次党代会精神，以科学发展观为指导，进一步增强忧患意识和使命意识，立足优化发展，围绕好中求进，以建设“三区四城”为目标，以创新型经济为主抓手，突出抓产业、上项目，提升城市品质，高度关注民生，为实现高水平崛起开好局、起好步。

-----立足优化发展，就是要坚定不移地推进产业结构调整，加快构建以现代产业体系为核心的创新型经济，加快提升自主创新能力，在产业建设上迈出坚实步伐。

-----围绕好中求进，就是要积极应对严峻复杂的发展形势，保持经济平稳健康可持续增长；加强和创新经济社会管理，最大限度减少不和谐因素，保持社会大局稳定。

-----提升城市品质，就是要围绕承接优质产业、吸引优秀人才、提高生活质量，进一步完善城市功能，优化空间布局，改善人居环境，使东莞成为创业热土、宜居城市。

-----高度关注民生，就是要强化执政为民宗旨，加快发展以民生为重点的各项社会事业，着力提升公共服务水平，办好十件民生实事。

综合考虑各方面因素，今年全市发展的预期目标是：全面完成珠三角规划纲要“四年大发展”各项目标任务，生产总值同比增长8%，人均生产总值增长7.5%，市财政一般预算收入增长10%，固定资产投资总额增长9%，社会消费品零售总额增长12%，外贸出口总额增长8%；现代服务业增加值占服务业增加值比重61.5%，先进制造业、高技术制造业增加值占规模以上工业增加值比重分别达到42.7%和29.5%，发明专利申请量和授权量分别增长12%和28%；城市居民人均可支配收入和农民人均纯收入增长8%，单位生产总值能耗下降4.25%，城镇登记失业率、居民消费价格涨幅分别控制在3%以内和4%左右。

围绕以上目标要求，重点抓好八个方面工作：

一、深入调整结构，切实推动产业建设

经济发展的根本动力在于产业。必须紧紧抓住产业建设这个关键，扎扎实实发展创新型经济。

加快培育壮大战略性新兴产业。着力引进大、高、精、尖项目，培育骨干企业，扩张产业规模，力争在新兴产业的重点领域、关键环节率先突破。加快省级薄膜太阳能光伏、物联网、半导体照明等产业基地建设，抓好宏威、中镓、华为等项目建设。实施“智慧东莞”工程，推进物联网和云计算运用，争取建立省战略性新兴产业云计算基地。探索政府引导、社会参与等方式，建立战略性新兴产业投资引导基金。

加快加工贸易和传统产业转型。深入推进全国加工贸易转型升级试点工作，落实“稳增长、调结构、促平衡”方针，继续实施外经贸“十个100”计划。落实促进优势传统产业发展和转型升级的指导意见，分行业制定具体实施方案。深入推进品牌带动战略，积极发展工业设计和动漫设计，推动企业技术、产品和管理升级。

加快打造现代服务业集聚板块。推进生产性服务业功能区建设，加快引进培育物流采购、客户服务、产品展示、资金结算等中心。推进中央商圈的整合提升，启动南城国际商务区建设，加快各镇中心区的服务产业集聚。研究出台发展楼宇经济优惠政策，积极引进地区总部和区域总部。继续办好电博会、漫博会、台博会等展会，力争将外博会升级为国家级博览会。探索制定鼓励电子商务、移动支付等新兴业态发展的政策，加快发展物流、金融、软件、新一代互联网、旅游休闲等产业。

二、强化创新驱动，集聚高端资源要素

进一步完善创新体系、集聚创新资源、优化创新环境，使科技创新成为转型升级的核心推动力。

促进科技、产业、金融有机结合。围绕培育新兴产业和提升优势产业，组织关键技术攻关，加快培育壮大一批创新型骨干企业。探索建立产业风险投资引导资金，大力引进风投、创投机构及种子基金，充分利用社会资本，加速孵化一批成长性创新企业。加快金融商务区和金融改革创新服务区建设步伐。积极推动松山湖高新区进入“新三板”扩容试点。在有条件的地区开展土地资本、金融资本、产业资本“三资融合”试点。建立企业专利动态数据库，支持企业开展专利权质押融资。鼓励发展中小企业担保机构、小额贷款公司等新型经济金融组织。

加强创新载体建设。加快组建国家半导体光源产品质量监督检验中心、华南设计创新园、北京大学光电研究院等公共创新平台。创新投入和管理机制，强化产权激励和绩效管理，充分发挥公共平台服务功能。加快散裂中子源项目建设。积极推动松山湖科技平台与各镇街企业对接合作。做大做强专业镇技术创新平台。引导有条件的企业设立技术中心等机构，创建一批国家级、省级企业研发平台。

优化科技创新环境。完善科技政策体系，加强科技专项资金管理，整合金融与社会资本，提高资金使用效率与引导功能。发挥政府牵线搭桥作用，促进产学研更紧密合作。积极发展技术评估、技术转移、科技信息等中介服务机构和行业协会组织，加快组建技术产权交易中心，促进科研成果应用转化。

实施人才强市战略。创新人才政策体系，有效发挥“人才东莞”专项资金作用。大力实施“十百千万”行动计划，积极引进培育创新科研团队、领军人才、紧缺人才和高技能人才。认真解决人才入户、配偶就业、子女入学等问题。重奖有突出贡献的杰出人才。推进留学人员创业园、博士后流动站等建设。采取校企合作、订单培训等方式，加快培养与产业发展相适应的技能型、实用型人才。

三、优化园区平台，引进培育龙头项目

发挥园区的产业集聚作用，着力实施大企业、大项目引领战略，进一步优化企业结构，提升产业集中度。

科学推进园区建设。进一步明确松山湖产业定位，吸引大项目和科研平台进驻。办好台湾高科技园，承接以色列产业园落户。加强虎门港的货源聚集，做好增值服务。科学定位生态园，完善基础设施和功能配套，争取引进一批高端企业。抓紧长安新区用海规划报批，加快土地资源整合。提升各镇街工业集聚区建设水平，增强大项目承载能力。严格筛选入园企业和项目，确保产出效益最大化。

瞄准优质项目重点招商。建立市级招商统筹协调机制，促进招商信息共享。落实重大项目快速反应机制，加强与知名投资中介的合作，集中力量引进大型央企、民企，引进日韩台等国家和地区的高科技项目。主动承接广州、深圳等城市的高端优质产业转移项目。加大土地统筹与闲置地盘活力度，科学安排用地指标。办好首届世界莞商代表大会。

加强龙头企业和上市公司培育。抓好总部企业认定，建立大企业储备库，筛选一批年产值10亿元以上的企业进行重点培育。加强政府服务，推动品牌企业、大型企业增资扩产。切实帮助企业解决土地产权等历史遗留问题，争取新增上市企业2—3家、年主营业务收入百亿元企业2家。

四、深化开放协作，确保经济平稳发展

积极应对外部环境挑战，高度重视帮扶企业和镇村发展，进一步加强区域合作，保障经济平稳发展。

不遗余力帮扶企业解困。坚持市镇领导走访重点企业制度，帮助解决实际困难，增强企业经营信心。规范镇村收费项目，积极争取省级收费减免。实施10亿元融资支持计划，帮助企业获得贷款和直接融资。积极争取用电指标，科学安排错峰用电，保障企业能源供给。加强劳务对接，缓解用工难题。优化中小企业服务，帮助搭建营销平台。

有效改善强化经济管理。落实对镇村的超收分成、贷款贴息、生态补偿等措施，减轻欠发达镇重大基建资金负担。逐步加大对农村的转移支付力度。安排1.39亿元推进市内“双到”扶贫，确保两个“80%”目标任务顺利完成。加强镇街政府性债务管理。完善农村集体资产管理实施办法，严控一般性支出和超前分配，减轻债务负担，防范经济风险。

多策并举促进内外均衡。认真落实省政府促进进口的二十七条意见。设立每年2亿元、连续3年的进口扶持资金，资助先进生产设备进口。开展“莞货全国行”活动。大力支持企业扩大内销市场，巩固欧美等传统市场，拓展东盟等新兴市场。落实促进民营经济发展上水平的实施意见，支持民营资本进入先进制造业、战略性新兴产业，实施“686”民营企业上市梯度培育工程、“323”高成长型中小工业企业培育计划，鼓励民营企业开展进出口业务和对外合作。

抢抓机遇深化区域协作。主动对接穗深港，从观念、交通、产业、市场和人才等方面，积极融入世界级城市群的发展。落实珠江口东岸地区紧密合作框架协议，加强深莞惠三市重大规划衔接，强化产业发展、交通运输、环境生态、社会公共事务等领域的合作。落实“双转移”战略，加快莞韶、莞惠产业转移园建设。全面完成韶关、云浮“双到”扶贫工作。做好对口支援新疆生产建设兵团农三师、西藏林芝、广西河池、重庆巫山等工作。

五、统筹城乡建设，提升城市承载能力

以重点项目建设为抓手，进一步完善基础设施，改善城乡环境，提升城市功能，持续增强城市承载力和辐射力。

突出城市功能提升。加快市民艺术中心、工人文化宫、篮球中心、网球中心、雅园新村二期等功能配套项目建设，积极配合穗莞深、莞惠城际轨道建设，推进市域轨道R2线、东莞新火车站建设和东莞东站升级改造。加快中央生态休闲区建设。加强地下空间综合开发。

突出设施水平提升。建成沿江高速、虎岗高速虎门港支线一期、东莞大道延长线、S120石排至桥头段。加快建设博深高速、从莞高速、虎岗高速延长线、虎门港支线二期和沿海公路。推进石大公路、东部快速企石至桥头段、S256南城至虎门段、S358长安至虎门段、S357樟木头至谢岗段的升级改造。加快梨川大桥、东平大桥建设。抓紧整治市主干公路堵塞点。建成110千伏及以上输变电工程10项。加快东江与水库联网供水二期工程，有效整合基层水厂，推进供水管网改造，建设水质质监网络，组建市水务投资集团。编制全市排水规划，加强城市内涝整治，启动127宗防灾减灾工程。

突出环境质量提升。落实用水总量控制、用水效率控制、水功能区限制纳污“三条红线”，实行最严格的水资源管理制度。加快截污次支管网建设，推进运河综合整治与内河涌治理。完善“三旧”改造政策及流程，健全土地出让金返拨机制，完善2万亩用地手续。加快垃圾处理厂的新建改造，推广生活垃圾分类处理，争取城镇生活垃圾无害化处理率达85%。加快7个环保专业基地建设。改造提升旗峰公园，基本建成黄牛埔和碧湖森林公园。推进生态景观林建设，实施退果还林工程。建成29个名村、300公里市级绿道。严格落实节能减排任务，深入开展清洁空气行动，加强空气质量PM值的监测。

六、推进社会建设，提高民生保障水平

全面发展社会事业，积极解决群众关注的难点热点问题，让全市人民更好地分享发展成果。

深化医疗体制改革。强化医疗卫生事业公益性质，明确政府补偿机制，抓好公立医院改革试点。落实社区卫生服务机构基本药物零差率销售，推动公立医院优先使用基本药物。健全基层医疗卫生服务体系，落实基本公共卫生和重大公共卫生服务项目。剥离镇街公立医院的疾病预防控制和卫生监督功能。组建市传染病后备医院。制定扶持中医药发展的具体措施。进一步完善医患纠纷调解机制。

提高社会保障水平。扩大社保覆盖面，推行全国统一的社保卡。出台临时救助办法。扩建市社会福利中心。将高龄津贴发放范围扩大至70岁以上户籍老人。加快残疾人服务设施建设。做好社会工作，设立扶持社会组织发展专项资金，创建20个社区综合服务中心示范点，规范购买社工服务制度。开展公益创投活动，办好第三届“东莞慈善日”。

激发就业创业活力。推广“村民车间”就业形式，帮助户籍劳动力特别是家庭困难人员就业。实施青年就业见习和培训计划，帮助高校毕业生就业。开展农民工专场招聘，做好新莞人就业服务。加快人力资源市场信息网络建设，建立就业信息收集发布制度。发挥小额贷款、创业奖励等的作用，激发社会创业活力。

全面发展民生事业。完善体育设施，推动群众体育发展。维护物价稳定，抓好“菜篮子”工程，兴建平价商店。加强房地产调控，防止过度发展房地产项目。拓宽商业贷款转公积金贷款业务，制定公租房管理办法，扩大保障房申请范围，有序推进农民公寓建设。优先发展公共交通，逐步推广新能源汽车，提高公交服务水平。稳定低生育水平，统筹人口发展，提高人口素质。继续做好国防人防、双拥共建、外事侨务、普法教育、民族宗教、统计审计、气象防灾、档案方志、科普、打私、工青妇幼等工作。

七、加强社会管理，有效维护安定和谐

坚持服务与管理并举，加大治理力度，理顺管理体制，不断提高社会管理水平，营造稳定和谐的社会环境。

强化社会治安综合治理。紧盯群众反映最强烈的治安问题，深入开展专项整治行动，严打多发性和恶性违法犯罪行为。坚持科技强警，完善公安指挥中心平台，加强联网报警和视频监控。逐步整合村（社区）治安联防队伍。加强商业区、车站码头、公园等重点场所的巡逻防控，提高街面见警率和出警效率，切实增强群众安全感。

加强公共安全管理。落实安全生产“一岗双责”，建立重大事故隐患监控信息系统。深化“三小”场所、人员密集场所等重点部位整治，打好“清剿火患”战役，筑牢消防安全“防火墙”。发挥综治信访维稳中心作用。加强矛盾纠纷调处化解和欠薪逃匿预警监控。深入开展“三打两建”行动和食品药品安全专项整治。强化应急管理与处置。

深化镇村体制改革。深入推进简政强镇改革，统一机构设置，规范权责关系。稳妥推进村级体制改革，探索推广社区政务服务中心。改革集体经济体制，探索推动从建设物业、分散收租向成立物业公司集中管理运营转变。

提升新莞人服务管理水平。加强新型社区建设，完善新莞人享受基本公共服务政策体系，提高就业培训、子女入学、医疗卫生等公共服务水平。落实居住证制度，强化出租屋管理。实施新莞人社会融入工程，开展商旅优惠、专列返乡等特色服务，增强城市认同感和归属感。

八、加快文化建设，着力提升城市文明

深化文化体制改革，强化社会主义核心价值体系建设，进一步提高市民素质，提升城市文明程度。

推动文化名城建设。完善文化专项资金使用管理办法。创建国家公共文化服务体系示范区，实施创建示范达标工程和文化惠民提升工程。编制历史文化名城保护规划，推动虎门销烟遗址等历史文化项目建设。出台文化产业扶持政策，积极发展创意设计等产业，培育文化龙头企业，支持本土传媒做大做强。扶持文化创作基地，打造特色文艺精品。统筹发展公益性文化事业，鼓励社会资本进入经营性文化服务领域。实施文化人才“三个100”工程。

推动教育体系完善。提高财政对教育拨款比例。加快学前教育改革发展，理顺幼儿园管理体制，每年投入1亿元补助各类幼儿园，年内新增公办幼儿园15所。推进公办中小学等级学校创建。加快职教城建设，建成高技能公共实训中心、东莞理工学校新校区、市技师学院，创建2—3所省级以上示范性中职学校。筹建酒店管理职业技术学院和光明职业技术学院，推进与香港理工大学合作办学，扶持本土高校发展。

推动城市文明提升。巩固全国文明城市地位，开展文明创建活动，加强爱国主义和理想信念教育，引导市民践行道德规范，弘扬尊老爱幼传统。加强未成年人思想道德建设，健全心理健康教育网络。广泛开展志愿服务活动。不断加强政府自身建设，坚持依法行政，提升政府服务，以良好政风带动社会风气转变、带动城市文明升华、带动发展步伐提速。

各位代表！团结凝聚力量，实干铸就伟业。我们在攻艰克难中成就了一个又一个东莞奇迹，也将在奋进拼搏中迎来一个又一个新的辉煌。未来的蓝图已经绘就，前进的号角再次吹响。让我们以邓小平理论和“三个代表”重要思想为指导，以科学发展观为统领，深入贯彻落实中央和省的决策部署，在中共东莞市委的领导下，团结和依靠全市人民，锐意创新，扎实进取，为加快转型升级、建设幸福东莞、实现高水平崛起而努力奋斗！
